# Book Reviews

**Published:** 1916-03

**Authors:** 


### BOOK REVIEWS

Books mentioned may be inspected at and ordered through this office. So far as possible, books received in any month will be reviewed in the issue of the second month following.

**Medical Clinics of Chicago,** Jan. 1916. Published bi-monthly by W. B. Saunders Co., Philadelphia, $8.00 per annum.

This publication includes a number of interesting clinical lectures, including such subjects as cerebro-spinal meningitis, infantile grip, hepatic cancer, trichinosis. It occupies a position midway between the typic high class journal and the text book.

**A Manual of Hygiene and Sanitation.** By Seneca Egbert, M. D. Professor of Hygiene and Dean of the Medico-Chirurgical College, Philadelphia. New (6th) edition, thoroughly revised. 12mo, 525 pages, with 141 figures and 5 plates. Cloth $2.25 net. Lea & Febiger, Philadelphia and New York, 1916.

It will be noted that this work includes both sanitation and personal hygiene. Ventilation is especially well presented for a work of the size. The charts showing the influence of filtration on typhoid in Philadelphia are particularly interesting. Some useful formulae are given for calculation of vital statistics. In general the work covers the subject quite thoroughly, though obviously, in a somewhat condensed form.

**A Treatise on the Principles and Practice of Medicine.** By Arthur R. Edwards, M. D., Professor of the Principles and Practice of Medicine and Clinical Medicine and Dean of the Northwestern University Medical School, Chicago. New (3d) edition, thoroughly revised. Octavo, 1022 pages, with 80 engravings and 23 full-page plates in colors and monochrome. Cloth, $6.00 net. Lea & Febiger, Philadelphia and New York, 1916.

While it is obvious that conspicuously novel points cannot be introduced into a work on practice without detracting from its usefulness, and while it can scarcely be claimed that there is any great lack of such works, this author has excelled ordinary standards in several ways. For instance, paratyphoid, tetany, various conditions of the saliva, certain entozoa and a number of other conditions, often omitted and usually merely touched on, are given a fair amount of space. The colored plates, 23 in number, are extremely useful, and while the book does not pretend to the completeness of specialized works in haematology, neurology, etc., one could really handle a case of blood disease or malaria pretty satisfactorily by reference to this book, whereas most works on practice deal with such diagnostic problems only far enough to be an aggravation. Credit is given Dr. I. P. Lyon for part of the work on the blood. We can scarcely agree with the statement that even small haematemeses are characteristically acid, or that the symptomatology of achylia gastrica bears any resemblance to that of hyperchlorhydria, except as any gastric case may present atypic or misleading symptoms, but we agree most heartily with the conservative statements as to the value of surgery in gastric ulcers.

**Surgical Operations with Local Anesthesia.** Second edition by Arthur E. Hertzler, A. M., M. D., Ph. D., F. A. C. S. 327 pages, 173 illustrations. Cloth bound, price $3.00. Surgery Publishing Company, New York.

The rapid sale of the first edition covering minor surgery and the demand for a more complete work upon the subject covering both major and minor surgical work, has induced Dr. Hertzler to present this second volume, which for completeness as to detail and price we believe places it in a class by itself among those text books upon this most interesting and growing subject. Dr. Hertzler's vast surgical experience and his work with Local Anesthesia particularly fits him as an authority upon this subject, and thus the second edition of his book places within the hands of the doctor a manual which for completeness and comprehensiveness, particularly recommends it. From a review of this book Dr. Hertzler seems to have overlooked no point of major or minor importance. The large number of illustrations clearly places up to the eye of the reader the text of the book and both the general practitioner and surgeon will appreciate this work as a reliable guide in their operation work under local anesthesia.

---

**Painless Child Birth, Eutocia and Nitrous Oxid-Oxygen Analgesia.** Carl Henry Davis, Chicago. Published by Forbes & Co., Chicago. 134 pages, $1.00.

This book reviews briefly the history of the attempt at producing painless labor and, comparing different methods, gives the preference to the anaesthetic noted in the title. A table shows the general mortality of labor to be ½%, reduced to about ¼% under favorable conditions.

---

**Text Book of Nervous Diseases,** in 30 Lectures. Robert Bing, Basel, translated by Charles L. Allen, M. D., Los Angeles; Rebman Co., 141-5 W. 36, N. Y. 111 illustrations, 481 pages, $5.00.

The work is logically divided into lectures on the peripheral nerves, dyskenesias, progressive muscular atrophies, cord diseases including hereditary ataxias, syphilogenic diseases, arterio-sclerosis of the nerve centers, acute infectious diseases affecting the same, diseases of the cerebrum and cerebellum, malformation, etc., dysglandular symptom-complexes, diseases of the sympathetic sytem including angio- and tropho-neuroses, epilepsy, psychoneuroses, migraine. An index of authors and a general index conclude the work. The work is well illustrated but not over illustrated. The author has presented the subject clearly and with a thorough understanding, and the translator, with the occasional addition of a foot-note regarding new matter, has contented himself with putting the original words of the author into good English.

---

**Transactions of the Tri-State Medical Association of the Carolinas and Virginia.** 17th Annual Session at Charleston, Feb. 17 and 18, 1915. Publication Committee: J. Howell Way, Waynesville, N. C., A. E. Baker, Charleston, S. C., C. M. Edwards, Richmond, Va.

The President, Dr. E. C. Register, editor of the Charlotte Medical Journal, in his address, dwells on the value of medical organizations and particularly emphasizes the need of open-mindedness in investigating any suggestion which may by any possibility lead to valuable results. The work of individuals, however valuable, dies with them unless communicated to proper scientific bodies and thus preserved. The scientific and clinical papers cover a variety of subjects and, in accordance with our precedent, we make no attempt to review them.

---

**Medical Alumni Catalogue,** University of Buffalo. Published by the Alumni Association.

2874 names are listed, 2737 graduates of the University of Buffalo, 137 of Niagara University prior to its amalgamation in 1898. 847 were known to be dead, 207 could not be traced, leaving a living membership of 1820. The credit of compilation, which was done by order of the Association, under the direction of its Executive Committee, is due to Miss Emma L. Chappell, College Secretary 1893-1913. The officers of the general and local branches, constitution and by-laws are included in the book. The work is divided into three parts: lists of classes, 1847-1915; alphabetic list of all alumni referring to the first part, except for the class of 1915; geographic list of living alumni by places of residence, except for the class of 1915. There are 723 physicians listed in the State Directory for Buffalo; 533 of the living alumni are listed for the same city. 365 are out of N. Y. State, many in foreign countries. Almost all of the alumni who could not be traced are graduates of classes before 1870 and the great majority are undoubtedly dead.

We trust that every alumnus will carefully examine the record and get into touch with his undergraduate acquaintances. It is also desirable that exact data should be filed regarding alumni who have not been traced or with regard to whom errors have been made. Three deaths, at least, have occurred since the compilation of the list in October.

---

**The Practical Medicine Series, 1915.** Chas. L. Mix, A. M., M. D., general editor, Year Book Publishers, 227 S. LaSalle St., Chicago. $10 for the annual series of 10 volumes.

Vol 8, Materia Medica and Therapeutics, Preventive Medicine, Climatology, Geo. F. Butler, Ph. G., A. M., M. D., Henry B. Favill, A. B., M. D., Norman Bridge, A. M., M. D., editors. 378 pages, $1.35 separately.

Vol. 9, Skin and Venereal Diseases, Oliver S. Ormsby, M. D., and Jas. Herbert Mitchell, M. D., editors; Miscellaneous Topics, Harold N. Moyer, editor. 240 pages, $1.35 separately.

Vol. 10, Nervous and Mental Diseases, Hugh T. Patrick, M. D., and Peter Bassoe, M. D., editors. 240 pages, $1.35 separately.

This series is the best practical review of current medical literature extant. Experience derived from the present war is noted in each of the above.

---

**A Text Book of Physiology: for Medical Students and Physicians.** By William H. Howell, Ph. D., M. D., Professor of Physiology, Johns Hopkins University, Baltimore. Sixth edition thoroughly revised. Octavo of 1043 pages, 305 illustrations. Philadelphia and London: W. B. Saunders Company, 1915. Cloth, $4.00 net; half morocco, $5.50 net.

It is scarcely necessary to state that a work that has become standard and has reached its sixth edition, is a good text book, in the field chosen. What impresses us most favorably is that this work is like certain employees who do little things not literally in the line of their immediate duty and yet without pre-empting the function of others. For example, the discussion of nerve regeneration, of the special senses, particularly the eye, of the nervous centers, of the blood, of blood pressure, of the emunctories, of caloric balance, of the chemistry of foods, etc, includes, quite unobtrusively hints of great value from extremely practical standpoints. The statement that rennin does not do anything but coagulate milk and the unbiased but critical statement of the arguments for and against specificity of such a ferment, afford a feeling of satisfaction after a long experience of putting one's finger on a fact and finding that it is not there. The book is equally valuable negatively. For example, our attention has lately been concentrated on blood.

It is practically valuable, although disappointing, to find that some of the elaborate genealogies of leucocytes contained in supposedly practical works in this line of diagnosis, are no better supported than some human genealogies. These items will illustrate why the work will prove of service to the practicing physician as well as to the student.

---

**Practical Cystoscopy and the Diagnosis of Surgical Diseases of the Kidneys and Urinary Bladder.** By Paul M. Pilcher, M. D., Consulting Surgeon to the Eastern Long Island Hospital. Second edition thoroughly revised and enlarged. Octavo of 504 pages, with 299 illustrations, 29 in colors. Philadelphia and London: W. B. Saunders Company, 1915. Cloth, $6.00 net; half morocco $7.50.

The author has comprehended his subject in a broad spirit. His book is not merely a description of a special technic, but enters into symptomatology from endocopic inspection and into pathology as is required for proper conception of underlying disease. He also discusses ureteral catheterization. The final chapters on the therapeutic use of the cystoscope and the use of the high frequency current also illustrate the same thoroughness. For these reasons, the book is valuable not only to the man who expects personally to use the cystoscope and allied instrments, but to the practitioner who wishes to underderstand the methods of diagnosis and treatment available.

---

**Post-Mortem Examinations.** By William S. Wadsworth, M. D., Coroner's Physician of Philadelphia. Octavo volume of 598 pages with 304 illustrations. Philadelphia and London: W. B. Saunders Company, 1915. Cloth, $6.00 net; half-Morocco $7.50.

The introduction and chapter on phenomena of death contain some particularly good suggestions, many of which are usually lacking in works of this scope, and some of which are novel. The discussion of mortuaries and instruments, and of general technic is also noteworthy. We need not dwell on the routine consideration of dissection of various parts and organs composing the body of the book. Medico-legal post mortems, causes of death, coroners' examinations, medical evidence, exhumation, embalming, toxicology, violence, especially shot wounds, photography, electricity, mensuration, repair of body, bibliography of useful books, are topics of unusual interest and including many details often omitted. The following table is so interesting and contains information so difficult to obtain that we quote it in full:

| Organ | Weight | Length | Breadth (or calibre) | Thickness | Specific gravity | Proportion to body |
|---|---|---|---|---|---|---|
| Brain | 1000-1700 | 0.16* | 0.13* | 0.12* | 1040 | 2% |
| Cord | 30* | 0.50 | 0.01 | 0.008* | | .5 |
| Glands | | | | all glands about | | |
| Mammary | 150-900 | 0.1* | 0.12* | 0.05* | 1050 | |
| Parotid | 20-30 | .... | .... | .... | 1050 | |
| Prostate | 15-35 | 0.25* | 0.35* | 0.02 | | |
| Sublingual | 2-4 | | | | | |
| Submaxillary | 7-10 | | | | | |
| Suprarenal | 5-10 | 0.05‡ | 0.03‡ | 0.005‡ | | |
| Thymus | 3-5 | | | | | |
| Thyroid | 25-40 | 0.03* | 0.06* | | | |
| Heart | 100-360 | 0.1* | 0.09‡ | 0.035‡ | 1050 | 0.5% |
| Aorta | 30-50 | .... | 0.017-0.03 | 0.0015* | 1070 | |
| Intestines | | | | | | |
| Duodenum | | 0.25 | 0.02 | | | |
| Small | 630-700 | 6-8 | | | 1040‡ | |
| Colon | 460-500 | 1.4-1.7 | 0.6‡ | | | |
| Liver | 1200-2000 | 0.2-0.35 | 15.‡ | 0.14‡ | 1050* | 2.5%* |
| Kidneys | 200-350 | 0.1-0.13 | 0.05-0.06 | 0.03-0.04 | 1050 | 0.45% |
| Lungs | 700-1300 | 0.26‡ | 0.16‡ | 0.14‡ | | |
| Ovary | 5-10 | 0.03* | 0.015* | 0.012* | | |
| Pancreas | 30-150 | 0.17* | 0.04* | 0.03* | 1045 | |
| Spleen | 100-200 | 0.1* | 0.08* | 0.03* | 1035* | 0.25% |
| Stomach | 125-175 | 0.25 | 0.1-0.14 | 0.006 | | |
| Esophagus | 25-50 | 0.2-0.3 | 0.04* | 0.008* | | |
| Testicle | 10-30 | 0.04* | 0.025* | 0.02‡ | 1040 | |
| Uterus | | | | | | |
| Virgin | 30-50 | 0.06* | 0.035* | 0.02* | 1050 | |
| Parous | 50-100 | 0.07* | 0.40* | 0.025* | | |
| Bladder | 30-60 | | | | | |

* Plus; ‡ Plus-Minus.

---

**Bone-Graft Surgery.** By Fred H. Albee, M. D., F. A. C. S., Professor of Orthopedic Surgery at the New York Post-Graduate Medical School and the University of Vermont. Octavo volume of 417 pages with 332 illustrations, 3 of them in colors. Philadelphia and London: W. B. Saunders Company, 1915. Cloth $6.00 net; half morocco $7.50 net.

This is one of the most highly specialized and individualized branches of surgery. Considerable space is devoted to the physiologic and pathologic principles upon which the art is dependent and it may surprise many to know that 10 general and 18 special uses of the graft are enumerated. Beside Pott's and other spinal disease, the method is applicable to the sacro-iliac joint, the tarsus, as a substitute for all sorts of metallic plates, screws and other retention devices for ordinary fractures, to produce permanent closure of foramin after nerve resection, to prevent slipping of the patella, to replace the head and neck of the femur and to reinforce a shallow receptaculum, to repair nasal deformities, those of the lower jaw, and to fill in cranial apertures and others. The only contraindications are markedly septic field or excessive scar tissue as an environment, though it is usually advisable to cure syphilis before attempting the graft. The electric motor outfit is described and the bulk of the work is a detailed consideration of the various problems involved in different bones and under different pathologic conditions.

